# Cost‐of‐illness analysis of chronic urticaria clinical management in five countries of Latin America

**DOI:** 10.1002/clt2.70016

**Published:** 2025-01-01

**Authors:** Jorge Sánchez, Leidy Álvarez, José Ignacio Larco, Luis Ensina, Guillermo Guidos‐Fogelbach, Cesar A. Reyes‐López, German D. Ramon, Karla Robles‐Velasco, Ivan Cherrez‐Ojeda

**Affiliations:** ^1^ Group of Clinical and Experimental Allergy (GACE) Hospital “Alma Mater de Antioquia” University of Antioquia Medellín Colombia; ^2^ Academic Group of Clinical Epidemiology (GRAEPIC) University of Antioquia Medellín Colombia; ^3^ Pharmacoeconomic Evaluation Group SURA Company, Colombia Medellín Colombia; ^4^ Allergy Department “San Felipe” Clinic Lima Peru; ^5^ Federal University of São Paulo São Paulo Brazil; ^6^ Research at Instituto Politécnico Nacional SEPI/ENMH México City Mexico; ^7^ Laboratorio de Bioquímica Estructural Instituto Politécnico Nacional Escuela Nacional de Medicina y Homeopatía Mexico City Mexico; ^8^ Instituto de Alergia e Inmunologia del Sur Bahia Blanca Argentina; ^9^ Institute for Allergology Charité – Universitätsmedizin Berlin Corporate Member of Freie Universität Berlin and Humboldt‐Universität zu Berlin Berlin Germany; ^10^ Universidad Espíritu Santo Samborondon Ecuador; ^11^ Respiralab Research Group Guayaquil Ecuador

**Keywords:** chronic spontaneous urticaria, cost of illness, diagnostic expenses, Latin America, therapeutic expenses

## Abstract

**Introduction:**

Chronic spontaneous urticaria (CSU) is a disease with a high impact on the quality of life of patients. There are some evaluations of the economic cost of the disease in developed countries, but there is little information about the economic cost of the disease in developing countries. Our aim was to assess the economic diagnostic and therapeutic expenses of CSU in five Latin American (LA) countries.

**Methods:**

A noninterventional multicenter cross‐sectional study was conducted in five LA countries: Brazil, Colombia, Ecuador, Mexico, and Peru. To determine the frequency of medical interventions as well as clinical and sociodemographic characteristics of CSU patients, questionnaires were administered to patients, primary care physicians, allergists, and dermatologists. In each country, diagnostics and therapeutic expenses were calculated by reviewing medical records, health insurance, and interviews. The main outcome was the yearly economic burden from the healthcare insurance perspective in each country.

**Results:**

According to the projected costs, Brazil had the highest urticaria cost per patient/year (7009.4 USD), followed by Mexico (3695.1 USD), Ecuador (3132.8 USD), Peru (2693.9 USD), and Colombia (2392.8 USD); the cost and the frequency of use of omalizumab and antihistamines explain the total cost differences between countries. Interventions such as medical visits and exams had similar costs between countries and represented less than 10% of total urticaria cost analysis in the five countries.

**Conclusion:**

The cost of the CSU in LA varies widely based on the health insurance coverage, the cost of the therapies, and the frequency of therapies used. Strengthening national health systems, as well as following the recommendations of international guidelines, seems to reduce the cost of CSU and improve the quality of patients.

## 1 INTRODUCTION

Chronic spontaneous urticaria (CSU) is a condition that affects between 0.5% and 1.2% of the population significantly impacting their quality of life.^1^, ^2^ CSU generates a loss of work, school, and leisure time. The primary treatment for this condition is antihistamines, but even with increased dosages, 40%–60% of patients do not achieve adequate control.^3^ Since the introduction of Omalizumab in 2014, many patients have recovered their quality of life, but the economic cost of the disease has increased significantly for the patients and national health systems. According to studies from the United States and Europe, the cost of CSU for health insurers is over 1 billion dollars per year.^4^, ^5^, ^6^, ^7^

Although international guidelines have proven to be effective for disease management,^3^, ^8^ some real‐word studies in Latin America (LA) suggest that urticaria specialists do not always follow these recommendations; in some countries, it is common to start omalizumab at lower doses than those recommended in international guidelines (150 mg/month over 300 mg).^9^, ^10^, ^11^, ^12^, ^13^, ^14^ Adherence to guideline recommendations is challenging in middle‐income countries because of the economic impact. In this study, we aimed to conduct a cost of illness (COI) analysis in different LA countries with diverse levels of per capita income, evaluate the direct expenses incurred by the disease and explore the EAACI/GA^2^LEN/EuroGuiDerm/APAAACI guideline compliance within each country.

## 2 METHODS

### 2.1 Characteristics of health systems in LATAM

In each of the five countries participating in the study, there is a health system that covers more than 80% of the population, with coverage of 94% in Colombia and 96% in Brazil. However, the type of procedures or medications that are covered and to which the patient has access varies in each country (Supplementary Material). The Colombian and Brazilian systems offer the highest coverage of medications for the management of urticaria, including the use of omalizumab, while in Ecuador and Peru, the public system usually does not cover this medication and in Mexico it does cover it but only in special cases. Among people who have a permanent job, 5%–12% also have a private health system; the highest figure for this private system was in Brazil (12%) and in Mexico (9%) and the lowest in Ecuador (4%). Through this system, some patients can access complementary therapies in Ecuador.

### 2.2 Study design

A cost of illness (COI) analysis was performed to calculate the project diagnostic and therapeutic expenses of CSU in five LA populations. The principal objective of the COI analysis was to estimate the yearly economic burden from the healthcare insurance perspective in Brazil, Colombia, Ecuador, Mexico, and Peru. Secondly, we compared the cost of every healthcare service between countries and the management of CSU in clinical practice. The costs considered were: (1) medical visits; (2) exams; (3) admission to Emergency/hospital; and (4) drugs (antihistamines, cyclosporine, Omalizumab and other treatments).

### 2.3 Data collection, healthcare resource and frequency of events

Five strategies were applied for the collection of the information.

1) An advisory board was convened, consisting of five key opinion leaders in the field from UCARE centers (Urticaria Care Excellent Centers https://ga2len‐ucare.com). The primary objective of the advisory board was to engage in in‐depth discussions regarding the practical implications of EAACI guideline recommendations within the unique contexts of each participating country. The specific topics covered during these discussions included, but were not limited to, the accessibility and availability of CSU interventions, preferences in therapeutic approaches, and the frequency of use of different treatment modalities based on the official information provided by every country's database.
2) Cost of interventions was calculated according to the official databases of each country. If the information was not available in the official databases, an open search was carried out (pages of non‐governmental organizations, hospitals, scientific societies). Colombia: SISMED https://www.sispro.gov.co/central‐prestadores‐de‐servicios/Pages/SISMED‐Sistema‐de‐Informacion‐de‐Precios‐de‐Medicamentos.aspx; IETS (Instituto de Evaluación Tecnológica en Salud); https://www.iets.org.co/ Peru: https://ietsi.essalud.gob.pe/; Mexico: https://www.gob.mx/salud; Ecuador: https://www.salud.gob.ec/; Brazil: https://redetsa.bvsalud.org/members/.
3) Information from each national reimbursement tariff was consulted to establish the cost of different interventions and to identify which of these therapies were covered by the health system, and which were not.
4) The frequency of ordering of each intervention by the doctor, and the frequency of use of each intervention by the patients, were verified through patient interviews and review of medical records.
5) The cost of each therapy was evaluated considering the different health insurers (private or public), the type of presentation, and whether the medication is patent or generic brand.

### 2.4 Ethics considerations

The ethics committee of the University of Antioquia approved this study (IN40‐2016) in accordance with the Helsinki Declaration of 1964 and its later amendments. All participating institutions gave their consent for the collection of information and the dissemination of the results.

### 2.5 Data analysis

The prevalence of CSU is unknown in most LA countries.^12^ Based on epidemiological data from other regions,^15^ the analysis considered a CSU prevalence of 1% (range from 0.5%–1.2%). The projected national cost of CSU per year was calculated using the same assumption. The current population in each country was determined using data from “Worldometer” (https://www.worldometers.info/world‐population) and cross‐referenced with local census.

The local currency was converted to American dollars for comparative purposes using the following exchange rates (2024'dollar January to June): Colombia $4400 = 1 USD, Peru $3.8 = 1 USD, Ecuador $1 USD = 1 USD, Mexico $20 = 1 USD, and Brazil $5 = 1 USD.

The cost of Emergency/hospital care was calculated by multiplying the median number of days by the daily cost. The cost of each drug was determined by multiplying the number of drugs used by the expense associated with managing a single drug administration. Concerning the visits and exams, costs for specialist visits and laboratory tests were taken into consideration.

## 3 RESULTS

### 3.1 Characteristics of CSU population and health insurances in each country

**FIGURE 1:**
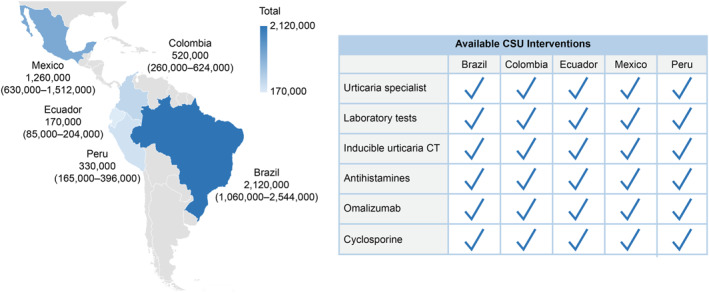
CSU population in LATAM and available interventions. CSU population in five LA countries with the assumption of a CSU prevalence of 1% (range 0.5%–1.2%). CSU interventions: Urticaria specialists (Dermatologist or Allergist), Laboratory tests (hemogram, Total IgE, anti‐TPO IgG, C‐reactive protein), and drugs (second‐generation antihistamines, omalizumab, and cyclosporine). CSU: Chronic Spontaneous Urticaria; IgE: Immunoglobulin E; anti‐TPO IgG: Immunoglobulin G antibodies against thyroid peroxidase.

**FIGURE 2:**
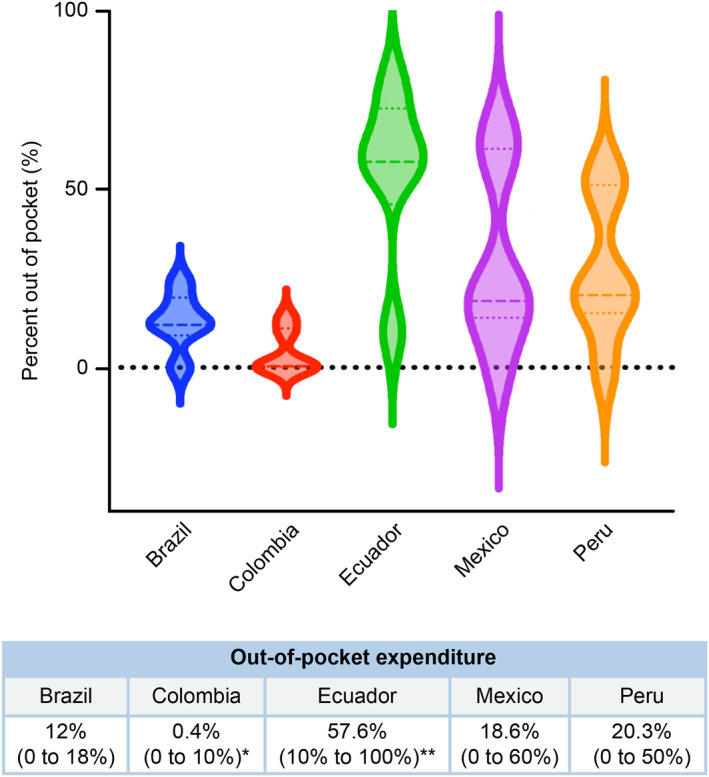
Distribution of patient out‐of‐pocket expenses in each country. Colombia had the lower expenses and Ecuador had the highest (*p* < 0.01)*, (*p* 0.04)** (Kruskal‐Wallis test).

With the assumption of a CSU prevalence of 1%, Brazil exhibited the highest number of CSU patients (Figure 1). Different healthcare services recommended by the CSU guidelines were available in all five countries. While all these countries provided health insurance that covered nearly 100% of the population, the out‐of‐pocket expenses for patients were significantly higher in Ecuador and lower in Colombia (Figure 2).

Other interventions used for some CSU patients, such as the inducible urticaria challenge test or the autologous serum skin test (ASST), were available across all five countries. However, in Brazil and Ecuador the coverage was limited to specific cases (Table S1). Antileukotrienes for CSU patients were covered by health insurances only in Colombia and Mexico.

### 3.2 Cost of urticaria intervention

The cost of healthcare services based on the step recommendation of the EAACI guidelines is presented in Table 1. The global spending of second‐generation antihistamines and cyclosporine was similar across the five countries (Tables S3 and S4). In Ecuador and Mexico, the costs of medical visits and laboratory tests (Hemogram, PCR, total IgE and anti‐TIPO IgG) were higher but not statistically significant (Table S5). In Ecuador and Brazil, the cost of cyclosporine and omalizumab was higher when compared to the other countries (Table 1).

**TABLE 1:** Cost of CSU interventions in 5 Latin American countries recommended by international guidelines (USD).

	Brazil	Colombia	Ecuador	Mexico	Peru	Mean cost
Medical visits	**25 USD (10–200)**	**15 USD (5–100)**	38 USD (10–120)	45 USD (40–100)	**25 USD (0–100)**	29 USD
Laboratory tests	17 USD (11–37)	21 USD (15–30)	57 USD (54–61)	**60 USD (43–125)**	**16 USD (10–93)**	34 USD
Antihistamines	0.84 USD (0.67–1.2)	**0.6 USD (0.05–1.1)**	**0.85 USD (0.8–1.2)**	**0.85 USD (0.8–2.3)**	0.7 USD (0.07–5.6)	0.76 USD
Cyclosporine (100 mg/capsule)	2.8. USD (1.8–2.6)	2 USD (1–3)	3.8 USD* (3.3–5)	2.5 USD (2.1–10)	**1.8 USD (0.49–2.4)**	2.5 USD
Omalizumab (150 mg/jeringe)	537 USD* (520–550)	**300 USD (280–320)**	420 USD (400–440)	470 USD (451–618)	380 USD (273–566)	441 USD
Emergency (per day)	350 USD (50–1000)	**400 USD (150–1000)**	125 USD* (50–200)	245 USD (83–425)	211 USD (160–330)	266 USD
Hospitalization (per day)	450 USD* (50–1000)	389 (250–2000)	**189 USD (100–500)**	244 USD (112–625)	211 USD (160–400)	296 USD
Inducible urticaria challenge test	109 USD (70–250)	**112 USD (75–200)**	100 USD (80–110)	108 USD (56.5–112.5)	60 USD* (40–78.9)	97 USD
Cost of other interventions						
Autologous serum skin test (ASST)	60 USD (40–150)	50 USD (39–98)	**30 USD (20–50)**	**75 USD (53–163)**	**30 USD (15–52.6)**	77 USD
Drug challenge test	130 USD (75–250)	150 USD (75–200)	240 USD (200–300)	**111 USD (83–160)**	**160 USD (60–395)**	158 USD
Food challenge test	120 USD (75–250)	150 USD (75–200)	**240 USD (200–300)**	**111 USD (83–160)**	160 USD (50–395)	156 USD

*Note*: The cost of each intervention was calculated according to the value charged to the insurers and/or individually in each country (median and range). In red is the highest cost and in green the lowest. * Significant differences in the cost (*p* < 0.05).

In Brazil, the cost for omalizumab was the highest, whereas in Colombia, it was the lowest (Table 1). The cost of a day in the emergency room was lowest in Ecuador (*p* = 0.03), while the cost of a day in the hospital was highest in Brazil (*p* = 0.04).

### 3.3 Frequency of interventions

Mexico and Peru had the highest frequency of medical visits (Table S2). All patients in the five countries underwent at least one round of laboratory tests. More than 70% of patients in all countries used antiH1 in higher than conventional doses and cyclosporine usage ranged from 10% to 25% across the countries. In Brazil, the utilization rate was the highest at 35% of CSU patients, whereas in the other countries it remained below 20%. Between 20% and 50% of patients who received omalizumab in Ecuador and Peru were on 150 mg/month. The number of patients admitted to emergency room or hospital was higher in Peru and lower in Colombia (Table S2).

### 3.4 CSU cost per year

**FIGURE 3:**
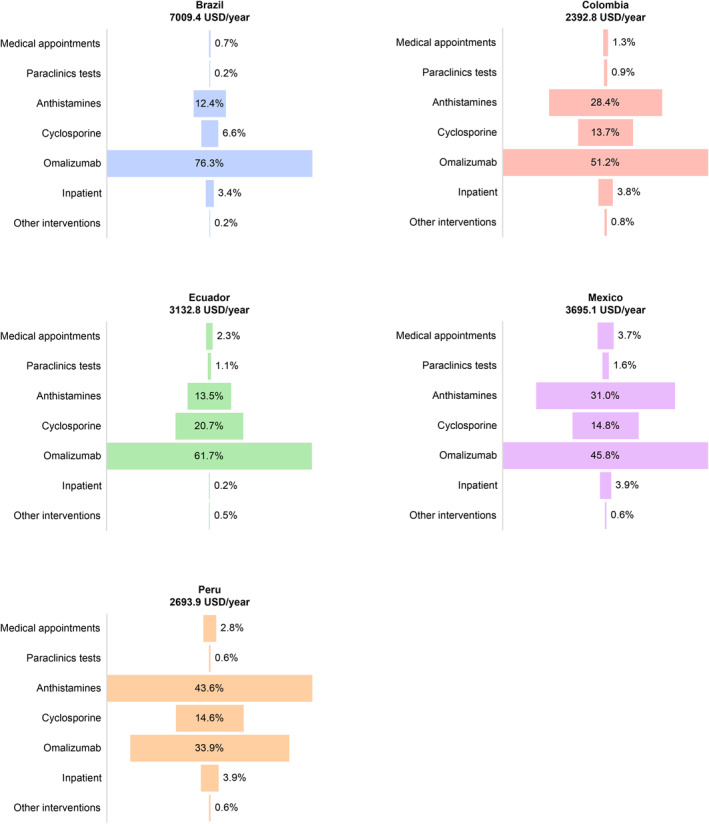
Total direct cost per patient/year according to the average cost of CSU management. The blocks show the percentage of each intervention according to the frequency of use in each country and its cost. CSU: Chronic Spontaneous Urticaria.

We calculated the cost of a CSU patient per year according to interventions proposed by the EAACI guideline steps and the medical management done in each country (Figure 3). The cost of illness may change enormously based on the severity of the disease in individual patients. The annual cost per patient/year was calculated based on the frequency of interventions by each country and ranged from 7009.40 USD in Brazil to 2398.80 USD in Colombia. The cost according to each step can be calculated using the information in Table 1. Based on the average income per person in each country (Table S6), the expenses derived from the management of the CSU with omalizumab were high, representing over 50% of the minimum monthly income in the five countries.

Drugs, especially antihistamines and omalizumab, were the major drivers of cost for patients in the five countries; the cost of using omalizumab in the five countries represented between 45% and 76% of the total annual patient costs. Other interventions (Urticaria specialists, laboratory tests) accounted for less than 10% of the total patient costs per year.

### 3.5 Compliance of CSU guideline recommendations

**FIGURE 4:**
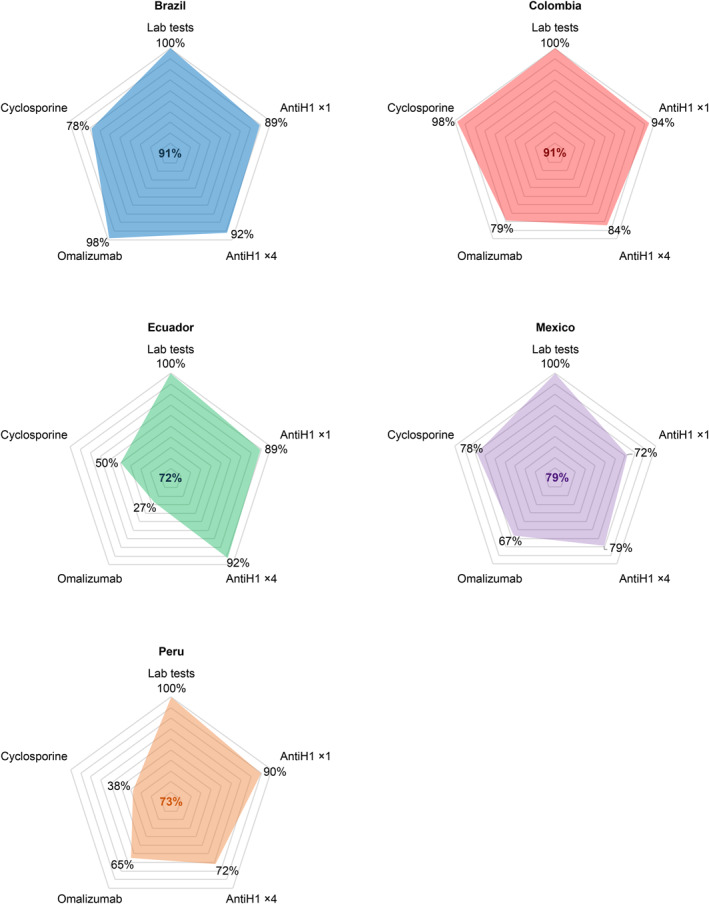
Compliance with CSU guideline recommendations. Compliance with CSU guideline recommendations; Laboratory tests (hemogram, Total IgE, anti‐TPO IgG, C‐reactive protein) should be performed at least once; Only second‐generation antihistamines should be used; omalizumab (300 mg/month) should be used in patients without clinical control after using antiH1 in high doses; cyclosporine after omalizumab. IgE: Immunoglobulin E; anti‐TPO IgG: Immunoglobulin G antibodies against thyroid peroxidase.

Based on data collected from health insurance and the review of at least 278 medical records in each country, we estimated compliance rates with guideline recommendations, according to control level based on the urticaria control test (UCT). Brazil and Colombia had the highest compliance rates and Peru and Ecuador had the lowest (Figure 4).

Despite varying frequencies, the primary causes of non‐compliance with the recommendations were similar across the countries. In cases where a standard dose of antihistamines was indicated, all patients received the drug. However, the main non‐compliance issue was the use of first generation antiH1. Similarly, when a higher dose of antiH1 was indicated, it was frequent that a first‐generation antiH1 was combined with a second‐generation one. Concerning omalizumab, the main failure was that some eligible patients did not receive it or received it at a lower dose than recommended by the guidelines. In Ecuador, Peru and Mexico, cyclosporine was indicated before omalizumab.

## 4 DISCUSSION

CSU is a disease with a high personal and social burden.^6^, ^12^, ^15^ While most patients achieve good disease control with relatively inexpensive and safe therapies like antihistamines,^16^ others require additional more expensive therapies such as omalizumab or cyclosporine.^17^, ^18^, ^19^, ^20^ These therapies offer significant relief to most patients but also come with a high economic cost. Studies have shown significant variations in the cost of CSU management across European countries, ranging from €1489 to €8142.^15^, ^21^ Our analysis is consistent with previous research, highlighting the high cost of managing CSU. This diseases' impact can be even more pronounced in countries with medium or low income, as is the case in most Latin American countries.^22^ Our study did not evaluate the cost of disabilities, which could further increase the economic impact for the health system and patients.

While most of the interventions recommended by the guidelines are available in the five countries studied, there are notable differences in their health systems, especially concerning out‐of‐pocket expenses for each patient. Medical appointments and laboratory screening for CSU are generally low‐cost practices in these countries. However, the cost of drugs, particularly omalizumab, stands out.

In Colombia, the out‐of‐pocket expenditure for patients is less than 1%, while in Ecuador it is over 50%. High out‐of‐pocket expenses can create significant challenges in achieving pharmacotherapy adherence and consequently in clinical control.^15^ This issue is especially pronounced in developing countries where the cost of the disease can account for more than 30% of patient's annual income, as observed in Colombia, Ecuador, and Peru. Colombia's income per person is lower than that of Brazil or Mexico and is similar to that of Ecuador or Peru. However, the extensive coverage provided by the health insurance in Colombia reduces the economic burden on patients, allowing better adherence to international guidelines.

Based on medical records, we observed that CSU severity was similar in all five countries, but there were significant variations in the frequencies of interventions used. The use of Omalizumab was more common in Brazil and Colombia due to the lower out‐of‐pocket costs for patients and cyclosporine in Ecuador and Peru. Nevertheless, the annual cost per patient was lower in Colombia than in Ecuador, Mexico, or Peru, where the use of omalizumab or second‐generation antihistamines is lower. This was because of the lower cost per unit of CSU drugs in Colombia compared to other countries in the region. In Colombia, regulations on omalizumab prices, combined with the efficient negotiation of treatment costs by healthcare centers serving millions of users, appear to have a beneficial effect, reducing the expenses and positively impacting the entire national health system.

Furthermore, improved access to medications and adherence to CSU guidelines appear to correlate with reductions in other indirect expenses. In Mexico and Peru, we observed higher rates of emergency room admissions and hospitalizations, which seem to be related to the elevated out‐of‐pocket expenses for patients. Other indirect expenses that could be increased by inadequate disease control include work absenteeism, presenteeism, and the development of comorbidities such as depression and anxiety. These supplementary costs should be taken into consideration in future studies as they can represent a significant financial burden for both patients and health insurers. In this study, we did not evaluate the use of systemic steroids as rescue therapy, and sometimes as control therapy although the latter use is not recommended. This aspect should be considered in future studies, considering that since it is an inexpensive therapy, its use may be high in countries with less ease of access to high‐cost therapies with an increased risk of adverse events.

Our study has some limitations. One of them is that we were not able to determine the out‐of‐pocket cost for each intervention. Therefore, we could not individually calculate for each intervention the cost it represents for the patient and for the health system (e.g. challenge tests, autologous serum test). However, for most therapies, especially antihistamines and omalizumab, this information could be obtained and since it represents the highest cost in management in all countries, the results represent the general picture. Another limitation of the study is that due to lack of information, some assumptions were made, such as the prevalence of urticaria in some countries. Despite this limitation with the information available on the cost of the different interventions, an analysis of different scenarios can be carried out that allows spending per country to be adjusted according to the prevalence obtained in future studies. The cost of urticaria can vary between the different populations studied according to the age of the patient, their geographical location (rural vs. urban), and other sociodemographic aspects. Although we were not able to make an evaluation including all these aspects, the evaluation carried out allowed us to identify the global cost of the disease in each country.

A strength of our study lies in the individual and comparative evaluation of the costs associated with different interventions in five LA countries. This approach not only helps us identify barriers to accessing CSU drugs in each country but also suggests possible solutions.

In conclusion, there is a great disparity in the management costs of urticaria in LA with significant economic and clinical implications for patients. Strengthening national health systems, as well as adhering to international guidelines recommendations appear to be effective strategies for reducing CSU costs and enhancing patient quality of life.

## AUTHOR CONTRIBUTIONS

**Jorge Sánchez**: Conceptualization; Investigation; Funding acquisition; Writing ‐ original draft; Methodology; Validation; Visualization; Writing ‐ review & editing; Formal analysis; Software; Project administration; Data curation; Supervision; Resources. **Leidy Álvarez**: Conceptualization; Investigation; Funding acquisition; Writing ‐ original draft; Methodology; Validation; Visualization; Writing ‐ review & editing; Resources; Supervision; Data curation; Software; Formal analysis; Project administration. **José Ignacio Larco**: Conceptualization; Investigation; Funding acquisition; Writing ‐ original draft; Methodology; Validation; Visualization; Writing ‐ review & editing; Software; Formal analysis; Project administration; Data curation; Supervision; Resources. **Luis Ensina**: Conceptualization, Investigation, Funding acquisition, Writing ‐ original draft, Methodology, Validation, Visualization, Writing ‐ review & editing. **Guillermo Guidos‐Fogelbach**: Conceptualization, Investigation, Funding acquisition, Writing ‐ original draft, Methodology, Validation, Visualization, Writing ‐ review & editing. **Cesar Reyes‐López A**: Conceptualization; Investigation; Funding acquisition; Writing ‐ original draft; Methodology; Validation; Visualization; Writing ‐ review & editing; Software; Project administration; Formal analysis; Data curation; Supervision; Resources. **German D. Ramon**: Investigation, Conceptualization, Funding acquisition, Writing ‐ original draft, Methodology, Validation, Visualization, Writing ‐ review & editing. **Karla Robles‐Velasco**: Investigation, Conceptualization, Funding acquisition, Writing ‐ original draft, Methodology, Validation, Visualization, Writing ‐ review & editing. **Ivan Cherrez‐Ojeda**: Conceptualization; Investigation; Funding acquisition; Writing ‐ original draft; Methodology; Validation; Writing ‐ review & editing; Visualization; Software; Formal analysis; Project administration; Data curation; Supervision; Resources.

## CONFLICT OF INTEREST STATEMENT

The authors do not have conflicts of interest with this topic.

## MEDICAL WRITING/EDITORIAL ASSISTANCE

No medical writing or medical assistance was requested for the manuscript.

## PRIOR PUBLICATION

This manuscript has not been previously published or presented in any form.

## POTENTIAL COMPETING INTERESTS

The authors report no competing interests.

## CONSENT TO PARTICIPATE

All participants involved in this study have provided informed consent before their participation in the study.

## CONSENT TO PUBLISH

We hereby confirm that consent to publish has been obtained from all participants. Participants were informed about the possibility of their data being published, and their consent was obtained accordingly.

## Supporting information

Supplementary Material

## Data Availability

The datasets generated and/or analyzed during the current study are available from the corresponding author on reasonable request. The data that support the findings of this study are not publicly available because they contain information that could compromise the privacy of research participants and data access in each case must be approved by the ethic committee. Requests for access must be directed to the corresponding author (JS) upon reasonable request for institutional submission.

## References

[1] Kolkhir P , Pogorelov D , Darlenski R , et al. Management of chronic spontaneous urticaria: a worldwide perspective. World Allergy Organ J. 2018;11(1):14. 10.1186/s40413-018-0193-4 29988758 PMC6030778

[2] Maurer M , Houghton K , Costa C , et al. Differences in chronic spontaneous urticaria between Europe and Central/South America: results of the multi‐center real world AWARE study. World Allergy Organ J. 2018;11(1):32. 10.1186/s40413-018-0216-1 30464782 PMC6238280

[3] Sánchez J , Zakzuk J , Cardona R . Evaluation of a guidelines‐based approach to the treatment of chronic spontaneous urticaria. J Allergy Clin Immunol Pract. 2018;6(1):177‐182.e1. 10.1016/j.jaip.2017.06.002 28709817

[4] O’Donnell BF . Urticaria: impact on quality of life and economic cost. Immunol Allergy Clin North Am. 2014;34(1):89‐104. 10.1016/j.iac.2013.09.011 24262691

[5] Weller K , Maurer M , Grattan C , et al. ASSURE‐CSU: a real‐world study of burden of disease in patients with symptomatic chronic spontaneous urticaria. Clin Transl Allergy. 2015;5(1):29. 10.1186/s13601-015-0072-9 26284152 PMC4538755

[6] Maurer M , Abuzakouk M , Bérard F , et al. The burden of chronic spontaneous urticaria is substantial: real‐world evidence from ASSURE‐CSU. Allergy. 2017;72(12):2005‐2016. 10.1111/all.13209 28543019 PMC5724512

[7] Kanters TA , Thio HB , Hakkaart L . Cost‐effectiveness of omalizumab for the treatment of chronic spontaneous urticaria. Br J Dermatol. 2018;179(3):702‐708. 10.1111/bjd.16476 29476533

[8] Zuberbier T , Abdul Latiff AH , Abuzakouk M , et al. The international EAACI/GA^2^LEN/EuroGuiDerm/APAAACI guideline for the definition, classification, diagnosis, and management of urticaria. Allergy. 2022;77(3):734‐766. 10.1111/all.15090 34536239

[9] Cherrez‐Ojeda I , Maurer M , Bernstein JA , et al. Learnings from real‐life experience of using omalizumab for chronic urticaria in Latin America. World Allergy Organ J. 2019;12(2):100011. 10.1016/j.waojou.2019.100011 30937137 PMC6439401

[10] Cherrez A , Maurer M , Weller K , Calderon JC , Simancas‐Racines D , Cherrez Ojeda I . Knowledge and management of chronic spontaneous urticaria in Latin America: a cross‐sectional study in Ecuador. World Allergy Organ J. 2017;10(1):21. 10.1186/s40413-017-0150-7 28546850 PMC5440895

[11] Wilches P , Wilches P , Calderon JC , Cherrez A , Cherrez Ojeda I . Omalizumab for chronic urticaria in Latin America. World Allergy Organ J. 2016;9(1):36. 10.1186/s40413-016-0127-y 27942350 PMC5120491

[12] Balp MM , Lopes da Silva N , Vietri J , Tian H , Ensina LF . The burden of chronic urticaria from Brazilian patients’ perspective. Dermatol Ther. 2017;7(4):535‐545. 10.1007/s13555-017-0191-4 PMC5698196 28748405

[13] Ensina LF , Valle SOR , Juliani AP , et al. Omalizumab in chronic spontaneous urticaria: a Brazilian real‐life experience. Int Arch Allergy Immunol. 2016;169(2):121‐124. 10.1159/000444985 27055122

[14] Ensina LF , de Lacerda AE , Machado LMdeO , Camelo‐Nunes I , Solé D . Long‐term omalizumab therapy for refractory chronic spontaneous urticaria: a real‐life experience. Ann Allergy Asthma Immunol. 2015;115(6):536. 10.1016/j.anai.2015.09.012 26653279

[15] Gonçalo M , Gimenéz‐Arnau A , Al‐Ahmad M , et al. The global burden of chronic urticaria for the patient and society. Br J Dermatol. 2021;184(2):226‐236. 10.1111/bjd.19561 32956489

[16] Sánchez J , Diez S , Cardona R . Clinical control of CSU with antihistamines allows for tolerance of NSAID‐exacerbated cutaneous disease. J Allergy Clin Immunol Pract. 2020;8(10):3577‐3583.e1. 10.1016/j.jaip.2020.06.057 32673879

[17] Wiuff AC , Knudsgaard Wiis MA , Heilskov S , et al. The impact of home treatment and self‐administration of omalizumab on chronic urticaria. World Allergy Organ J. 2022;15(12):100725. 10.1016/j.waojou.2022.100725 36531648 PMC9731880

[18] Melé‐Ninot G , Serra‐Baldrich E , Spertino J , et al. Are antihistamines still used during omalizumab treatment for chronic spontaneous urticaria? Eur J Dermatol. 2022;32(5):629‐631. 36468733 10.1684/ejd.2022.4334

[19] Sánchez‐Caraballo JM , Tamayo‐Quijando LM , Velásquez‐Lopera MM , et al. Consenso para el manejo práctico de la urticaria en atención primaria. Acta Médica Colombiana [Internet]. 2023;48(1). [cited 2023 Nov 7]. 10.36104/amc.2023.2722

[20] Sánchez J , Alvarez L , López JF . Indication of omalizumab for chronic urticaria using the “urticaria control test” instead of “urticaria activity score”: possible impact for health systems. Actas Dermosifiliogr. 2023;S0001‐7310(23). 00079‐0. 10.1016/j.ad.2022.10.044 36754254

[21] Shaker M , Oppenheimer J , Wallace D , et al. Optimizing value in the evaluation of chronic spontaneous urticaria: a cost‐effectiveness analysis. J Allergy Clin Immunol Pract. 2020;8(7):2360‐2369.e1. 10.1016/j.jaip.2019.11.004 31751758

[22] Arias‐Cruz A , González‐Díaz SN , Macías‐Weinmann A , et al. [Quality of life in chronic urticaria and its relationship with economic impact and disease control in patients attended to at the University Hospital of Monterrey, Mexico]. Rev Alerg Mex. 2018;65(3):250‐258. 10.29262/ram.v65i3.398 30176203

