# Supplementary material for: Cost‐of‐illness analysis of chronic urticaria clinical management in five countries of Latin America
Source: Clin Transl Allergy. 2025 Jan 1;15(1):e70016. doi: 10.1002/clt2.70016 (PMC11693417; doi:10.1002/clt2.70016)
Supplement: Supplementary file 1 — Supplementary Material [file CLT2-15-e70016-s001.docx]

**Title:**  **Cost-of-illness analysis of chronic urticaria clinical management in five countries of Latin America.**

**Authors:** Jorge Sánchez MD ^1^, Leidy Álvarez MD ^1,2,3^, José Ignacio Larco MD ^4^, Luis Ensina MD ^5^, Guillermo Guidos-Fogelbach MD ^6^, Cesar A Reyes-López MD ^7^, German D Ramon MD ^8^, Karla Robles-Velasco MD^9,10^, Ivan Cherrez-Ojeda M.Sc. ^9,10^.

**Affiliation:**

1. Group of Clinical and Experimental Allergy (GACE), Hospital “Alma Mater de Antioquia”, University of Antioquia, (Medellín, Colombia).
2. Academic Group of Clinical Epidemiology (GRAEPIC), University of Antioquia, (Medellín, Colombia).
3. Pharmacoeconomic evaluation group, SURA Company, Colombia. (Medellín, Colombia).
4. Allergy Department, “San Felipe” Clinic, (Lima, Peru).
5. Federal University of São Paulo, (São Paulo, Brazil).
6. Research at Instituto Politécnico Nacional, SEPI/ENMH. (México city, Mexico).
7. Laboratorio de Bioquímica Estructural, Instituto Politécnico Nacional, Escuela Nacional de Medicina y Homeopatía, (Mexico City, Mexico).
8. Instituto de Alergia e Inmunologia del Sur, (Bahia Blanca, Argentina).
9. Universidad Espíritu Santo, (Samborondon, Ecuador).
10. Respiralab Research Group, (Guayaquil, Ecuador).

**Corresponding authors:**

Jorge Sanchez, Allergology Department, University of Antioquia. Address Cra 27 n 37 B sur 69 (Medellín-Colombia) Phone +57 300 3934000. E-mail: [jorgem.sanchez@udea.edu.co](mailto:jorgem.sanchez@udea.edu.co)

Ivan Cherrez-Ojeda, Universidad Espiritu Santo. Address: Km. 2.5 via Samborondon. Phone +59345114555. E-mail: [ivancherrez@gmail.com](mailto:ivancherrez@gmail.com)

**SUPLEMENTAL MATERIAL**

**Table S1. Costs of medical attention and paraclinical tests**

| ***Cost of interventions*** | | | | | |
| --- | --- | --- | --- | --- | --- |
|  | **Brazil** | **Colombia** | **Ecuador** | **Mexico** | **Peru** |
| **Medical attention** |  |  |  |  |  |
| *Allergist visit* | 10 to 200 USD | 5 to 100 USD | 10 to 120 USD | 40 to 100 USD | 0 to 100 USD |
| *Dermatologist visit* | 10 to 300 USD | 5 to 100 USD | 10 to 80 USD | 35 to 100 USD | 0 to 150 USD |
| *Emergency (per day)* | 50 to 1000 USD | 150 to 1000 USD | 50 to 200 USD | 83 to 425 USD | 160 to 330 USD* |
| *Hospitalization (per day)* | 50 to 1000 USD | 250 to 2000 USD | 100 to 500 USD | 112.5 to 625 USD | 160 to 400 USD* |
| **Paraclinical tests** |  |  |  |  |  |
| *Hemogram* | 2 to 5 USD | 1.2 to 4 USD | 9 to 10 USD | 3.8 to 8 USD | 0 to 14 USD |
| *PCR* | 4 to 10 USD | 1.25 to 4 USD | 8.5 to 10 USD | 4 to 10 USD | 0 to 36 USD |
| *IgG TPO* | 1 to 12 USD | 10 to 14 USD | 20 to 21.6 USD | 15 to 25 USD | 0 to 23 USD |
| *IgE total* | 4 to 10 USD | 3 to 8 USD | 16 to 20 USD | 20 to 83.3 USD | 0 to 20 USD |
| *ASST* | 40 to 150 USD | 39 to 98 USD | 20 to 50 USD | 53 to 163 USD | 0 to 52.6 USD |
| *Inducible urticaria CT* | 70 to 250 USD | 75 to 200 USD | 100 USD | 56.5 to 112.5 USD | 0 to 78.9 USD |
| *Medication CT* | 75 to 250 USD | 75 to 200 USD | 200 to 300 USD | 83 to 160 USD | 0 to 395 USD |
| *Food CT* | 75 to 250USD | 75 to 200 USD | 200 to 300 USD | 83 to 160 USD | 0 to 395 USD |

**Note:** * Significant differences in the cost (*p* <0.05).

**Table S2. Frequency of use of interventions per patient.**

| Event frequency per patient | | | | | |
| --- | --- | --- | --- | --- | --- |
| Interventions | **Brazil** | **Colombia** | **Ecuador** | **Mexico** | **Peru** |
| Medica visits (Median and range) | 2 (1 to 10) | 2 (1 to 4) | 3 (2 to 7) | 5 (4 to 15) | 4 (2 to 10) |
| Emergency assistance (patients per year) | 25% | 23% | 6% | 40% | 50% |
| Emergency (Day per event) | 2 (1 to 5) | 1 (1 to 2) | 1 (1 to 3) | 1 (1 to 3) | 1 (1 to 5) |
| Hospital assistance (patients per year) | 0 to 10% | 0 to 5% | 1 to 3% | 20% | 5% |
| Hospital (Day per event) | 3 to 10 days | 1 to 4 days | 1 to 3 days | 1 to 3 days | 0 to 5 days |
| Round of conventional paraclinical tests (per year) | 1 to 3 | 1 to 2 | 1 to 3 | 0 to 3 | 0 to 5 |
| Round other paraclinical tests (per year) | 0 to 2 | 0 to 1 | 1 to 2 | 0 to 5 | 0 to 3 |
| Pharmacotherapy patients’ frequency |  | | | | |
| *AntiH1 2^nd^ generation conventional dose* | 21% | 29% | 18% | 11% | 12% |
| *AntiH1 2^nd^ generation higher dose* | 79% | 71% | 82% | 89% | 88% |
| *Cyclosporine* | 15%  (10% to 17%) | 15%  (10% to 18%) | 25%  (15% to 30%) | 20%  (13% to 25%) | 14%  (6% to 18%) |
| *Omalizumab patients* | 35%  (30% to 50%) | 17%  (15 to 20%) | 12%  (10% to 30%) | 15%  (10% to 30%) | 10%  (10% to 30%) |
| *Omalizumab and cyclosporine* | 5% | 3% | 5% | 10% | 0% |

**Note:** Conventional paraclinical tests (Hemogram, total IgE, anti-TPO IgG, PCR); no conventional paraclinical tests (ASST, inducible urticaria challenge test, drug challenge test, food challenge test)

**Table S3. Cost of medication.**

| **Drugs** | **Brazil** | **Colombia** | **Ecuador** | **Mexico** | **Peru** |
| --- | --- | --- | --- | --- | --- |
| *Loratadine tablet* | 0.25-0.78 USD | 0.05 to 1 USD | 0.05 to 1.5 USD | 0.13 to 0.86 USD | 0.01 to 1.05 USD |
| *Cetirizine tablet* | 1.1-1.3 USD | 0.05 to 1.25 USD | 0.88 to 1.20 USD | 0.32 to 1.8 USD | 0.02 to 5.16 USD |
| *Desloratadine tablet* | 0.89-1.23 USD | 0.05 to 1.25 USD | 0.76 to 0.95 USD | 0.16 to 2.1 USD | 0.02 to 11.02 USD |
| *Levocetirizine tablet* | 0.82 – 1.02 USD | 0.05 to 1.25 USD | 0.70 to 1.05 USD | 0.46 to 1.86 USD | 0.04 to 5 USD |
| *Rupatadine tablet* | No available | 0.05 to 1.30 USD | 1.10 to 1.14 USD | 2.3 to 2.93 USD | 0.31 to 10 USD |
| *Fexofenadine tablet* | 1.31 – 2.26 USD | 0.05 to 1.55 USD | 1.1 to 1.39 USD | 0.5 to 4.21 USD | 0.14 to 7.1 USD |
| *Bilastine tablet* | 0.49 – 0.8 USD | 0.05 to 1.25 USD | 1.1 to 1.39 USD | 1.9 to 2.4 USD | 0.26 to 12.6 USD |
| *Ketotifen tablet* | No available | 0.2 to 0. 75 USD | No available | 0.11 to 0.66 USD | 0.05 to 4.4 USD |
| *Difenidramine tablet* | No available | 0.2 to 0.75 USD | No available | 0.12 to 0.33 USD | No available |
| *Hidroxicine tablet* | 0.25 – 0.37 USD | 0.2 to 0.95 USD | 0.05 to 0.1 USD | 0.32 to 1.38 USD | No available |
| *Cyclosporine capsule 100 mg* | 2.8 USD | 1 to 3 USD | 3.39 to 5 USD | 2.1 to 10 USD | 0.46 to 2.4 USD |
| *Omalizumab (jeringe 150 mg)* | 637 USD | 280 to 320 USD | 400 to 440 USD | 451.6 to 618 USD | 273 to 566 USD |
| *Omalizumab (jeringe 75 mg)* | No available | 240 to 300 USD | No available | 309 to 316 USD | No available |
| *Systemic sterods (daily dose)** | 0.4 USD | 0.1 to 0.4 USD | 0.58 to 1 USD | 1 to 4 USD | 0.05 to 4.74 USD |
| *Montelukast 10 mg* | 0.32 – 0.72 USD | 0.50 to 1.2 USD | 0.8 to 1.25 USD | 0.5 to 2.2 USD | 0.1 to 1.2 USD* |

**Note:** *Reference of systemic steroids; prednisone for 20 mg.

**Table S4. Cost of medications in pediatric presentation.**

| **Drugs** | **Brazil** | **Colombia** | **Ecuador** | **Mexico** | **Peru** |
| --- | --- | --- | --- | --- | --- |
| *Loratadine syrup bottle* | 0.06 – 0.13 USD (ml) | 0.02 to 0.2 USD (ml) | 0.2 to 0.3 USD (ml) | 0.02 to 0.19 USD (ml) | 0.03 to 0.02 USD (ml)* |
| *Cetirizine syrup bottle* | 0.14 USD (ml) | 0.3 to 0.5 USD (ml) | 0.3 to 0.4 USD (ml) | 0.07 to 0.29 USD (ml) | 0.3 to 0.4 USD (ml)* |
| *Desloratadine syrup bottle* | 0.11 – 0.15 USD (ml) | 0.1 to 0.2 USD (ml) | 0.5 1.4 USD (ml) | 0.09 to 0.25 USD (ml) | 0.1 to 0.3 USD (ml)* |
| *Levocetirizine syrup bottle* | 0.1 to 0.2 USD* | 0.15 to 0.2USD (ml) | 0.8 to 0.6 USD (ml) | 0.05 to 0.35 USD (ml) | 0.2 to 0.24 USD (ml)* |
| *Rupatadine syrup bottle* | No available | 0.17 USD (ml) | No available | 0.39 to 0.42 USD (ml) | 0.3 to 0.4 USD (ml)* |
| *Fexofenadine syrup bottle* | 0.11 USD (ml) | 0.12 to 0.18 USD (ml) | 0.2 to 0.42 USD (ml) | 0.12 to 0.42 USD (ml) | 0.2 to 0.3 USD (ml)* |
| *Bilastine syrup bottle* | 0.13 USD (ml) | No available | 0.5 USD (ml) | 0.4 to 0.43 USD (ml) | 0.3 to 0.5 USD (ml)* |
| *Ketotifen syrup bottle* | 0.04 USD (ml) | 0.02 to 0.03 USD (ml) | 0.1 to 0.2 USD (ml) | 0.03 to 0.1 USD (ml) | 0.1 to 0.2 USD (ml)* |
| *Difenidramine syrup bottle* | No available | 0.05 to 0.15 USD (ml) | No available | 0.02 to 0.11 USD (ml) | No available |
| *Hidroxicine syrup bottle* | 0.07 – 0.11 USD (ml) | 0.05 to 0.15 USD (ml) | No available | 0.07 to 0.3 USD (ml) | No available |
| *Montelukast 5 mg* | 0.43 – 0.72 USD (ml) | 0.3 to 0.5 USD (ml) | 0.4 to 0.9 USD (ml) | 0.09 to 0.79 USD (ml) | 0.09 to 0.6USD (ml)* |
| *Montelukast 4 mg* | 0.43 – 0.72 USD (ml) | 0.35 to 0.4 USD (ml) | 0.4 to 0.9 USD (ml) | 0.08 to 0.5 USD (ml) | 0.05 to 0.6 USD (ml)* |

**Note:** The minimum and maximum value are presented in each cell in dollar according convention to the median value 2022: Colombia $4400 = 1 USD, Peru $3.8 = 1 USD, Ecuador; $1 = 1 USD, Mexico $20 = 1 USD, Brazil $5 = 1 USD.

**Table S5. Frequency of intervention requirement.**

| **Event frequency per patient** | | | | | |
| --- | --- | --- | --- | --- | --- |
| **Drugs** | **Brazil** | **Colombia** | **Ecuador** | **Mexico** | **Peru** |
| Allergist visit (per year) | 2 to 10 | 1 to 4 | 2 to 7 | 6 to 15 | 2 to 10 |
| Dermatologist visit (per year) | 1 to 3* | 0 to 2 | 2 to 5 | 4 to 12 | 2 to 10 |
| Other medical visit (per year) | 0 to 1 | 0 | 2 to 4 | 8 to 15 | 0 to 4 |
| Emergency assistance (patients per year) | 20 to 30% | 23% | 2 to 10% | 40% | 50% |
| Emergency (Day per event) | 3 to 10 | 1 | 1 to 3% | 10 to 30 | 0 to 5 |
| Hospitalization assistance (patients per year) | 0 to 10% | 0 to 5% | 1 to 3% | 20% | 5% |
| Hospitalization (Day per event) | 3 to 10 | 1 to 4 | 1 to 3 | 1 to 3 | 0 to 5 days |
| Paraclinics per patients |  |  |  |  |  |
| *Hemogram (per year)* | 1 to 3 | 1 to 2 | 1 to 2 | 2 to 4 | 0 to 5 |
| *PCR (per year)* | 1 to 4 | 1 to 2 | 1 to 4 | 1 to 2 | 0 to 5 |
| *IgG TPO (Per year)* | 1 to 2 | 1 to 2 | 1 to 2 | 0 to 2 | 0 to 5 |
| *IgE total (per year)* | 1 to 3 | 1 to 2 | 1 to 2 | 1 to 3 | 0 to 5 |
| *ASST (per year)* | 0 to 1 | 0 | 1 to 2 | 0 to 5 | 0 to 2 |
| *Inducible urticaria CT (per year)* | 0 to 2 | 1 | 1 to 2 | 1 to 4 | 0 to 3 |
| *Drug CT (per year)* | 0 to 2 | 1 | 1 | 1 to 3 | 0 to 3 |
| *Food CT (per year)* | 0 to 2 | 1 | 1 | 1 to 3 | 0 to 3 |
| Pharmacotherapy patients’ frequency |  |  |  |  |  |
| *AntiH1 first generation x1* | 0 | 0 | 10% | 10% | 1% |
| *AntiH1 first generation x2* | 0 | 0 | 2% | 10% | 1% |
| *AntiH1 first generation x3* | 0 | 0 | 0 | 0 | 4% |
| *AntiH1 first generation x4* | 0 | 0 | 0 | 0 | 2% |
| *AntiH1 second generation x1* | 20 | 30% | 20% | 10% | 12% |
| *AntiH1 second generation x2* | 30 | 15% | 10% | 40% | 50% |
| *AntiH1 second generation x3* | 0 | 5% | 20% | 50% | 10% |
| *AntiH1 second generation x4* | 50 | 50% | 50% | 0 to 10%* | 20% |
| *Cyclosporine* | 15% | 10 to 15% | 25% | 20% | 0 |
| *Cyclosporine dose in children <12 years* | 3 mg/kg/day | 3 mg/kg/day | 3 mg/kg/day* | 2.5 mg/kg/day | 2.5 mg/kg/day |
| *Cyclosporine dose in patients >12 years* | 3 mg/kg/day | 3 mg/kg/day | 3 mg/kg/day* | 2.5 mg/kg/day | 2.5 mg/kg/day |
| *Omalizumab patients* | 50% | 15 to 20% | 30% | 30% | 30% |
| *Omalizumab dose* | 300 mg/4w | 300 mg/4w | 300 mg/4w | 300 mg/4w | 300 mg/4w |
| *AntiH1 first and second generation* | 0 | 0 | 20% | 40% | 15% |
| *Omalizumab and cyclosporine* | 5% | 3% | 5% | 10% | 0% |
| *Loratadine patients* | 5% | 5 to 10% | 5% | 10% | 2% |
| *Cetirizine patients* | 0 | 15 to 20% | 10% | 20% | 5% |
| *Desloratadine patients* | 0 | 20 to 30% | 5% | 50% | 2% |
| *Levoceterizine patients* | 40% | 5 to 15% | 5% | 60% | 5% |
| *Rupatadine patients* | 0 | 5 to 15% | 15% | 40% | 20% |
| *Fexofenadine patients* | 15% | 15 to 25% | 80% | 60% | 25% |
| *Bilastine patients* | 40% | 15 to 25% | 25% | 40% | 35% |
| *Ketotifen patients* | 0 | 0 | 2% | 0 | 1% |
| *Diphenydramine patients* | 0 | 0 | 3% | 0 | No apply |
| *Hydroxyzine patients* | 0 | 0 | 20% | 0 | No apply |

**Table S6. Sociodemographic characteristics**

| **Sociodemographic characteristic** | | | | | |
| --- | --- | --- | --- | --- | --- |
| **Interventions** | **Brazil** | **Colombia** | **Ecuador** | **Mexico** | **Peru** |
| Monthly minimum wage | 264 USD | 400 USD | 450 USD | 345 to 519 USD | 269.7 USD |
| Median monthly income per person | 548 USD | 400 USD | 450 USD | 410 USD | 420.5 USD |
| Monthly cost for a patient of the public insurance | 0 to 2000 USD | 0 to 1000 USD | 0 to 1000 USD | 175 to 2500 USD | 0 to 56.6 USD |
| Monthly cost for a patient of the private insurance | 11 to 320 USD | 100 to 2000 USD | 30 to 400 USD | 125 to 2000 USD | 26.3 to 184 USD |

**Note:** Absenteeism secondary to urticaria by illness or medical consultation
